# The application of blended learning in obstetrics teaching: a comprehensive teaching model integrating an intelligent, interactive induction platform

**DOI:** 10.3389/fmed.2025.1670126

**Published:** 2025-12-09

**Authors:** Kaifen Gu, Haiyan Pan, Meiyuan Dong, Xuanjin Chen, Huiping Yuan

**Affiliations:** 1 Department of Obstetrics, Zhoushan Hospital, Zhoushan, Zhejiang, China; 2 Department of Public Health, Zhoushan Hospital, Zhoushan, Zhejiang, China; 3 Department of Nursing, Zhoushan Hospital, Zhoushan, Zhejiang, China; 4 University College London, London, United Kingdom; 5 Department of Director’s Office, Zhoushan Hospital, Zhoushan, Zhejiang, China

**Keywords:** diversified blended teaching learning, medical intelligent induction interaction platform, nursing teaching, student nurse, obstetrics

## Abstract

**Introduction:**

Hybrid learning, integrating diverse teaching methods and resources, is promising in education. In nursing, it suits varied learning styles via multiple channels and offers time-place flexibility, aiding busy practice nurses. Information and Communication Technology (ICT) is vital in modern nursing education. Medical intelligent induction platforms, as its form, simulate real-life scenarios for safe skill practice and provide real-time feedback for learning enhancement.

**Objective:**

This study aimed to evaluate impact of blended learning in obstetrics teaching using a medical intelligent induction interactive platform on multiple dimensions. These dimensions include the scores of theoretical teaching and practical operation, independent learning ability, comprehensive ability, and overall nursing quality.

**Methods:**

From August 2022 to August 2023, 70 practice nurses in our hospital were selected, and they were divided into the control group and the research group. The control group received training and teaching through conventional methods. The research group adopted a blended learning model using the medical intelligent induction interactive platform.

**Results:**

The research group outperformed the control group significantly (*P* < 0.05) in theoretical teaching performance, practical operation performance, independent learning ability, comprehensive ability, and overall nursing quality. Moreover, the 35 trainee nurses in the research group showed relatively high overall satisfaction with the blended learning model combined with the medical intelligent guidance interactive platform.

**Conclusion:**

Integrating the blended learning method with the medical intelligent induction interactive platform for teaching clinical intern nurses can significantly enhance their relevant nursing knowledge, practical operation skills, and autonomous learning ability, thereby elevating the quality of nursing.

## Introduction

Nursing, as a crucial applied science, plays an indispensable role in the overall operation and development of hospitals. It closely adheres to the hospital’s quality policy and quality objectives, serving as the fundamental support for ensuring the quality of medical services and enhancing patient satisfaction (1). Clinical nurses, as the direct executors of nursing work, the proficiency of their nursing skills and practical abilities directly affect the treatment outcome and recovery process of patients. Therefore, possessing solid and comprehensive nursing skills is an essential quality for every clinical nurse (2).

Internship nurses, as the new force in the nursing team, the cultivation of their nursing knowledge and practical abilities is of great significance for the sustainable development of the nursing profession (3). Conducting regular and systematic training on nursing content for internship nurses is the main way to improve their nursing knowledge and practical abilities (4). Through scientific and reasonable training, it can help them quickly adapt to the clinical working environment, combine theoretical knowledge with practical operations closely, and thus provide better quality nursing services for patients (5).

Among all the clinical departments, the obstetrics department has its own uniqueness. It is a high-risk and highly specialized department. The obstetrics department encounters many critical cases, and the physical condition of pregnant women is constantly in a state of change (6). Any slight oversight in observation or improper handling could directly endanger the life safety of both the mother and the baby (7). Obstetric expertise is not only extensive but also highly diverse, covering various aspects from prenatal care, the delivery process to postpartum care. Professional skills also require a great deal of practical operation to be mastered proficiently, such as monitoring the labor process and performing neonatal resuscitation (8). However, when intern nurses enter the obstetric internship, they often find that due to the forgetting of previous learning content and the dispersion of the knowledge system, they feel inadequate when facing actual work. This requires experienced teachers to impart professional knowledge and to systematically organize, summarize and expand it to help them build a complete and clear obstetric knowledge framework (9).

However, the current traditional training model for obstetric intern nurses has many drawbacks. Due to the relatively short internship period and the fact that the traditional training methods mainly consist of classroom lectures and simple operational demonstrations, lacking interactivity and personalization, the training outcomes vary greatly (10). The intern nurses participating in the training often only have a superficial understanding of the relevant knowledge and struggle to deeply grasp its essence. When facing various complex cases, they are unable to flexibly and accurately apply the knowledge they have learned to actual nursing operations, thus greatly reducing its practicality (11).

With the rapid development of information technology, its application in the field of nursing education has become increasingly widespread and profound, bringing about unprecedented changes to nursing learning (12, 13). In nursing education, the application of information technology has greatly enriched teaching resources and learning methods. For instance, virtual simulation technology can simulate real clinical scenarios, allowing trainee nurses to repeatedly practice in a virtual environment, thereby improving their operational skills and ability to handle emergencies (14); online learning platforms have broken the limitations of time and space, enabling trainee nurses to access abundant learning materials at any time and place, and conduct self-study and interactive communication (15). Studies have shown that the application of information technology in nursing education can effectively cultivate various key skills of trainee nurses, including autonomous learning ability, critical thinking ability, teamwork ability, and information literacy (16). The blended learning, as a teaching method that combines online and offline learning, has become an inevitable trend for many fields to address the challenges of digitalization and has played a crucial role in medical education (17). It fully leverages the convenience of online teaching and the interactivity of offline teaching, and can flexibly choose the appropriate teaching method according to different teaching contents and learning objectives, providing learners with a more personalized and diverse learning experience (18). As a new direction for the reform of medical nursing teaching in China, vigorously developing diverse blended learning models is the key to improving the teaching effectiveness and quality of clinical nursing. Through blended learning, it can stimulate the learning interest and initiative of trainee nurses, enhance their learning engagement, and thus enable them to better master nursing knowledge and skills (19).

As an online service platform for sharing medical content resources, the Medical Intelligent Induction Interactive Platform, with its powerful functions, provides strong support for clinical nursing teaching (20). This platform can offer online cloud learning services, allowing trainee nurses to study the course content independently according to their own time and pace (21). At the same time, it can also assign tasks and provide feedback. Teachers can promptly understand the learning situation of trainee nurses and give targeted guidance and suggestions. By applying the Medical Intelligent Induction Interactive Platform, the quality of nursing education can be effectively improved, the interest of nurses in learning can be stimulated, and their autonomous learning ability and innovative thinking can be cultivated (22).

Based on this, this study combined blended learning with the medical intelligent induction interaction platform to construct a joint teaching model, aiming to provide more efficient and high-quality nursing teaching for intern nurses and conduct in-depth analysis of its application effects, with the expectation of providing new ideas and methods for the training of obstetric intern nurses, and further improving the quality and level of obstetric nursing teaching.

## Materials and methods

### General data

From August 2022 to August 2023, based on the relevant inclusion criteria (namely, trainee nurses engaged in clinical work in our hospital), a total of 70 trainee nurses were selected to participate in the training and teaching work of this study. To allocate the participants, they were assigned to either the control group or the research group sequentially based on their order of entry into the department. This process resulted in 35 nurses being allocated to each group.

The basic information of the two groups of trainee nurses was statistically analyzed. The results are shown in Table 1. After comparison, no statistical difference was found (*P* > 0.05), and the two groups were comparable.

**TABLE 1:** Comparison of general data of trainee nurses in both groups.

Groups		Control group (*n* = 35)	Research group (*n* = 35)	*t*/χ^2^	*P*
Gender (male/female)		2/33	1/34	0.348	0.555
Age (years)		21.81 ± 0.92	21.77 ± 1.04	0.171	0.865
Education background	Junior college	18	20	0.230	0.631
	Undergraduate	17	15	0.230	0.631

### Sample size calculation

The sample size was calculated by the changes of the Competency Inventory for Nursing Students (CINS) score (23). By setting α = 0.05, β = 0.10, a dropout rate of 10%, the number of nurses in this study were 70 using power and sample size calculation (PS) Software.

### Description and nature of the medical intelligent induction interactive platform

The medical intelligent induction interactive platform used in this study is a comprehensive online teaching service platform based on Internet technology. It is highly professional and targeted, integrating a wealth of medical care teaching resources and leveraging advanced information technology means to provide a highly interactive and functionally rich learning environment for teachers and students.

The platform has an extensive library of nursing course resources, covering various knowledge contents in different nursing disciplines such as basic nursing, specialized nursing (including obstetric nursing, internal medicine nursing, surgical nursing, etc.), nursing management, and nursing ethics. The resources come in diverse forms, including detailed, teaching courseware (PPT), as well as vivid and intuitive teaching videos and 3D animations.

This platform has powerful interactive features, enabling real-time communication and interaction between teachers and students, as well as among students themselves. Teachers can use the platform to issue learning tasks, assign homework, and organize online discussions; students can ask questions to teachers at any time, submit homework, participate in discussions, and share their learning experiences and insights with other classmates. Additionally, the platform features a learning group function, allowing students to join different learning groups based on their interests and learning needs, and conduct in-depth learning exchanges and cooperation within the groups.

The platform can provide personalized learning suggestions and resource recommendations based on students’ learning situations and needs. By analyzing data on students’ online learning behaviors, such as study time, progress, and correct answer rates, the platform can understand students’ learning characteristics and weak points, and then recommend suitable learning content based on their learning levels and interests, helping students achieve personalized learning.

The platform has intelligent management functions, which enable teachers to manage teaching and students’ learning conveniently. Teachers can easily post course information, manage student lists, grade assignments, and calculate students’ academic performance; students can conveniently view course schedules, learning progress, assignment requirements, and promptly understand their own learning situations. At the same time, the platform also has a facial recognition sign-in function, which can accurately record students’ attendance and improve the efficiency and accuracy of teaching management.

### Teaching method

#### Composition of teaching team

The teaching team members included: 2 deputy chief nurses, 2 specialist nurses, 5 chief nurses and 2 nurses; among them, three teachers were hired by Jilin Medical University. They all have teaching qualifications and relevant teaching experience. Teachers in the fields of psychology, language, behavior, laws and regulations, and university awareness are all 4 head nurses of our hospital. They mainly designed relevant training courses for the nursing department and implemented them in the onboarding training.

#### Course design

The control group received training and teaching through conventional methods. The trainee nurses in our hospital were required to conduct regular pre-reading before class; during the class, the teacher explained the relevant content and asked random questions; after class, the trainee nurses could complete the exercises independently and discuss with the teacher and other trainee nurses to find the relevant answers and appropriate methods to solve the problems they didn’t understand.

The research group adopted a blended learning model and combined with the medical intelligent induction interactive platform. Before the implementation of the blended learning, the relevant training and teaching team developed an assessment project, conducted a content assessment related to nursing, and recorded the assessment results after the teaching. This teaching model was divided into three stages: pre-class learning, classroom teaching, and post-class assessment.

(1) Pre-class learning: Utilizing the resource library of the medical intelligent induction interactive platform for nursing courses, the teacher edited relevant nursing content through PPT, videos, and 3D animations, integrates and uploaded nursing-related examination questions, allowing trainee nurses to study online independently, thereby obtaining rich nursing knowledge and improving learning efficiency. The relevant nursing content was arranged both online and offline. Offline: Practical tasks such as scenario simulation and role-playing were assigned. Online: Trainee nurses completed the learning of relevant nursing knowledge and discussed questionnaires, gave reasons, previewed the relevant content of the next course, and marked out the difficult points. Trainee nurses uploaded the marked questions and difficult points to the study group, and the teacher checked them immediately, which shortened the offline observation time and directly answered the questions and difficulties of the trainee nurses.

(2) Classroom teaching: In the classroom, the intern nurses used the platform for facial recognition sign-in, which saved the time consumed by traditional roll call. The teacher played online videos for the intern nurses to watch. After the viewing, relevant questions were raised and the intern nurses were asked to answer. An interesting and quick solution method was adopted to enhance the learning enthusiasm of the intern nurses. Moreover, by watching operation videos, the intern nurses got simulations in the actual classroom, enabling them to be more immersed in the current environment and better improve their practical operation skills and comprehensive abilities.

(3) Post-class assessment: The online platform learning situation and offline practical performance of the intern nurses were evaluated through a scoring system, which aimed to stimulate the intern nurses’ own initiative in learning and further improve the teaching quality. Additionally, after completing the learning tasks and scoring, the intern nurses raised their own questions and concerns. The teacher understood and recorded the content raised by the intern nurses or answer these questions in class, enabling the intern nurses to more clearly recognize their own shortcomings and strive to improve them. The teacher uploaded the offline classroom content to the platform, and the intern nurses watched it during their spare time and followed the video for operations. Through the three-dimensional guidance of images, text and sound, the intern nurses’ autonomous learning ability could be better enhanced.

### Measurement outcomes

#### Performance of the nursing knowledge

The performance of the nursing knowledge of the intern nurses was evaluated. The content of the evaluation results was divided into two dimensions: theoretical teaching and practical operation, with a total score ranging from 0 to 100 for each dimension. The theoretical knowledge covered: disease knowledge and the process of the nursing teaching system; the practical operation covered: basic operations and nursing professional operations. These two dimensions were set up in an objective and structured manner for the evaluation sites. The higher the score, the better the performance of the intern nurses.

#### Autonomous learning ability assessment

The self-learning ability scoring scale was used to evaluate the self-learning ability of practice nurses. The scale covered three dimensions: self-management ability, learning cooperation ability and information ability, with a total of 28 items. Likert 5-level scoring method was adopted, ranging from “completely inconsistent” to “completely consistent,” with a score ranging from 0 to 5 points and a total value ranging from 28 to 140 points. The higher the score, the stronger the independent learning ability of practicing nurses was proved.

#### Comprehensive ability score

The core competencies and clinical practice levels of the trainee nurses were evaluated using the CINS and the mini-clinical evaluation exercise (mini-CEX) scoring scales (23, 24). The CINS scale covers six dimensions: clinical biological science dimension, general clinical skills dimension, critical thinking and reasoning ability dimension, caring dimension, ethics and responsibility dimension, and lifelong learning dimension. The total score ranges from 38 to 190, and the higher the score, the higher the comprehensive ability of the trainee nurses. The CINS scale had a Cronbach’s alpha of 0.98. The mini-CEX scale covers seven dimensions: medical interview, physical examination, communication skills, clinical judgment, humanistic care, organizational efficiency, and overall performance. The total score for each dimension ranges from 0 to 9, and the total score is 63. The higher the score, the stronger the clinical practice ability of the trainee nurses. The Cronbach’s alpha of mini-CEX scale was 0.90.

#### Quality of nursing performance

The quality of nursing after the interns’ training was evaluated. The assessment covered aspects such as communication and collaboration, nursing operations, emergency response capabilities, and service attitude. The total score for each aspect was 100 points, and the higher the score, the better the nursing quality.

### Statistical analysis

SPSS 31.0 statistical software was used for analysis and processing. Kolmogorov-Smirnov test was applied to check whether the data met the normal distribution, which was represented by ($\bar{x}\pm s$), the homoticity of variance test was performed by levene method, the comparison between two groups was performed by independent sample *t*-test, the comparison within groups was performed by paired *t*-test, the counting data was described by [n (%)], and the comparison between groups was performed by x^2^ test. *P* < 0.05 was statistically significant.

## Results

### Comparison of post-study results between the two groups of practice nurses

As shown in Table 2, the theoretical teaching and practical operation scores of the research group were significantly higher than those of the control group, with statistical differences (*P* < 0.05).

**TABLE 2:** Comparison of post-study results between the two groups of practice nurses ($\bar{x}\pm s$).

Groups	Cases (n)	Theory teaching (points)	Practical operation (points)
Control group	35	88.80 ± 2.30	90.00 ± 2.16
Research group	35	96.29 ± 1.18	96.63 ± 0.81
*t*		−17.149	−17.035
*P*		0.006	0.004

### Comparison of scores of independent learning ability of practice nurses between the two groups

As shown in Table 3, the scores of self-management ability, learning cooperation ability and information ability of the research group were significantly higher than those of the control group, with statistical differences (*P* < 0.05).

**TABLE 3:** Comparison of scores of independent learning ability of practice nurses in two groups ($\bar{x}\pm s$).

Groups	Cases (n)	Self-management ability (points)	Learning cooperation ability (points)	Information ability (points)	Total score (points)
Control group	35	28.457 ± 3.24	27.09 ± 3.09	26.00 ± 1.61	81.54 ± 5.59
Research group	35	39.40 ± 2.199	32.00 ± 2.07	28.94 ± 3.18	100.34 ± 3.97
*t*		−16.537	−7.813	−4.885	−16.221
*P*		0.012	0.010	0.013	0.027

### Comparison of comprehensive ability scores of practice nurses between the two groups

As shown in Table 4, the CINS and mini-CEX scores of the research group were significantly higher than those of the control group, with statistical differences (*P* < 0.05).

**TABLE 4:** Comparison of comprehensive ability scores of practice nurses in two groups ($\bar{x}\pm s$).

Groups	Cases (n)	CINS (points)	Mini-CEX (points)
Control group	35	151.51 ± 6.36	50.57 ± 3.49
Research group	35	163.91 ± 4.76	58.09 ± 1.92
*T*		−9.235	−11.162
*P*		0.018	0.014

### Comparison of overall nursing quality of practice nurses between the two groups

As shown in Table 5, the scores of communication and cooperation, nursing operation, emergency response ability and service attitude of the research group were significantly higher than those of the control group, with statistical differences (*P* < 0.05).

**TABLE 5:** Comparison of overall nursing quality of practice nurses in two groups ($\bar{x}\pm s$).

Groups	Cases (n)	Communication and collaboration (points)	Nursing operations (points)	Emergency response capacity (points)	Service attitude (points)
Control group	35	81.06 ± 5.56	87.14 ± 2.49	77.69 ± 5.35	83.60 ± 4.24
Research group	35	93.57 ± 3.24	94.60 ± 1.82	93.86 ± 3.20	94.03 ± 2.66
*t*		−11.511	−14.320	−15.355	−12.315
*P*		0.017	0.024	0.023	0.018

### Evaluation of diversified blended learning model combined with medical intelligent induction interactive platform in experimental group

As shown in Table 6, 35 trainee nurses in the research group were evaluated, and their satisfaction with the teaching method combining the blended learning model with the medical intelligent guidance interactive platform was analyzed. The overall satisfaction was relatively high.

**TABLE 6:** Evaluation of the teaching mode of hybrid learning combined with medical intelligent induction interactive platform by practice nurses in the research group.

Groups	Very satisfied	Satisfied	Generally satisfied	Dissatisfied
Liking smart sensing blended learning	24 (68.57)	8 (22.86)	3 (8.57)	0 (0.00)
Abundant classroom teaching resources	22 (62.86)	13 (37.14)	0 (0.00)	0 (0.00)
Improving learning enthusiasm	23 (65.71)	11 (31.43)	1 (2.86)	0 (0.00)
Improving problem solving skills	24 (68.57)	10 (28.57)	1 (2.86)	0 (0.00)
Improving the ability to analyze problems	21 (60.00)	12 (34.29)	2 (5.71)	0 (0.00)
Upgrading nursing skills	25 (71.43)	9 (25.71)	1 (2.86)	0 (0.00)
Enhancing nursing knowledge	23 (65.71)	12 (34.29)	0 (0.00)	0 (0.00)
Improving communication skills	24 (68.57)	11 (31.43)	0 (0.00)	0 (0.00)
Strengthening team spirit	22 (62.86)	13 (37.14)	0 (0.00)	0 (0.00)

## Discussion

The results of this study show that the scores of the research group in theoretical teaching performance, practical operation performance, independent learning ability, comprehensive ability, and overall nursing quality were significantly higher than those of the control group. At the same time, the overall satisfaction of 35 trainee nurses in the research group toward the teaching method combining the blended learning model with the medical intelligent guidance interactive platform was relatively high. These results suggest that a teaching model that combines blended learning with the intelligent interactive platform for medical guidance demonstrates superiority over the traditional teaching model in multiple key aspects, effectively enhancing the comprehensive quality and nursing quality of trainee nurses, thereby verifying the feasibility and effectiveness of this teaching model.

With the changes in the age structure of the population and the increasing proportion of the elderly, people’s demands for maternal and infant health have become more precise, and the requirements for care have been continuously upgraded (25). At the same time, the clinical demand for the professional ability and practical knowledge of trainee nurses has also been increasing (26). The blended learning model, which combines online and offline teaching methods, has been proven to enhance the teaching quality and learning interest of nursing interns (27). The significant improvement in the theoretical and practical performance of the research group in this study was in line with this view, further demonstrating the advantages of the blended learning model in the field of nursing education. Consistently, Zhong et al. suggested that in the flipped classroom with a blended learning process of histology practical, enhancing the quality of online learning boosts student satisfaction and improves knowledge learning; peer-to-peer interactions and instructor-to-peer interactions in the physical classroom improved knowledge construction (28).

The medical intelligent induction interaction platform, as a shared resource and online teaching service platform in the field of medical care, relevant studies have indicated that it can significantly enhance the learning interest of trainee nurses and improve the overall quality of nursing education through the rapidity and convenience of the network (29). In this study, the outstanding performance of the research group in independent learning ability, comprehensive ability, and overall nursing quality is closely related to the platform’s role in stimulating learning interest, promoting knowledge sharing and communication. The platform’s abundant resources, powerful interactive functions, and personalized learning support provide a more superior and efficient learning environment for trainee nurses, helping them better master nursing knowledge and skills (30).

The CINS and Mini-CEX scores are commonly used indicators for evaluating the core competencies and clinical practice of trainee nurses. In this study, the scores of the research group significantly improved. This was due to the diverse mixed teaching methods that provided standardized online and offline training for the trainee nurses. During the training process, teaching methods such as scenario simulation, role-playing, and group cooperation fully stimulated the learning motivation of the trainee nurses, enabling them to shift from passive learning to active learning and actively participate in practical activities, thereby effectively enhancing their core competencies and clinical practice abilities. The medical intelligent induction interactive platform enables the sharing of nursing knowledge and other contents, allowing trainee nurses to obtain relevant professional knowledge immediately (31). Through the establishment of study groups, it facilitates free communication and team learning. This learning method promotes the communication and collaboration skills of trainee nurses within the team, significantly enhancing their comprehensive abilities, and thereby improving the quality of nursing in subsequent internships. The combination of blended learning and the medical intelligent induction interaction platform enables the teaching staff to obtain the information of the trainee nurses online, understand their specific needs, and conduct targeted teaching. This maximizes the benefits of the trainee nurses during the learning process, clarifies the teaching content and objectives, and effectively improves various dimensions of nursing quality such as communication and cooperation skills, nursing operation skills, emergency response capabilities, and service attitude (32, 33).

### Limitations

However, this study has some limitations that may affect the interpretation and generalization of the results. Firstly, the sample size in this study is relatively small and the study period is restricted. A small sample size may not comprehensively represent the characteristics of the entire population of trainee nurses. This lack of representativeness raises concerns about the applicability of our research results to a broader group. In the future, it is imperative to expand the sample size and conduct multi-center, large-scale studies. This would enable a more precise analysis of the teaching model’s effects and provide a more robust reference for clinical practice.

Secondly, there are issues regarding the validation of the methods used in the study, particularly in terms of controlling for potential confounding factors. The impact of teaching instructors was not thoroughly evaluated. Different instructors possess distinct teaching styles and varying levels of professional expertise. These differences can significantly influence the learning outcomes of intern nurses. The previous experience of the intern nurses was also not adequately considered as a confounding factor. Nurses with different levels of practical experience and knowledge bases may have different acceptance levels of the teaching methods and subsequently achieve different learning outcomes. Future research should design more rigorous schemes to control and adjust for these potential confounding factors. This could involve stratifying the sample based on prior experience or using statistical methods to account for these variables, thereby enabling a more accurate assessment of the independent effect of the teaching model.

Furthermore, there are limitations in the generalizability of our data interpretation and conclusions. This research was conducted in a single hospital. Different hospitals vary in terms of medical resources, teaching environments, and management models. These differences can have a significant impact on the implementation effect of the teaching model. As a result, the findings of our study may not be directly applicable to other hospitals with different conditions. Our study focused on a specific nursing field and did not deeply explore the application effect of this teaching model in different specialized nursing education. Different nursing specialties have unique requirements and learning needs. The teaching model that works well in one specialty may not be as effective in another. Therefore, future research should be carried out across hospitals and different specialties to further explore the wide application prospects of this teaching model. This would provide more targeted guidance for different nursing education scenarios and enhance the overall quality of nursing education.

## Conclusion

By integrating the blended learning method with the medical intelligent induction interactive platform to conduct teaching for clinical intern nurses, it can significantly enhance the relevant nursing knowledge, practical operation ability, and autonomous learning ability of the intern nurses, improve their overall capabilities, and thereby enhance the quality of nursing and learning satisfaction.

## Data Availability

The original contributions presented in this study are included in the article/supplementary material, further inquiries can be directed to the corresponding author.
